# Laparoscopic Nephrolithotomy in a Horseshoe Kidney

**DOI:** 10.7759/cureus.7099

**Published:** 2020-02-25

**Authors:** Mohamed O Breish, Siddharth Sarnaik, Seshadri Sriprasad, Musaab Hamdoon

**Affiliations:** 1 Urology, Darent Valley Hospital, Dartford, GBR; 2 Urology, Canterbury Christ Church University, Canterbury, GBR

**Keywords:** laparoscopic surgery, nephrolithotomy, nephrolithiasis, horseshoe kidney., anomalous kidneys, renal stones

## Abstract

Horseshoe kidney (HSK) is the common renal fusion congenital anomaly, affecting about 0.25% of the global population. Although most HSKs are detected incidentally, they may present with clinical findings, including urinary tract infections (UTI), stone formation, and obstruction. Nephrolithiasis, observed in 20% of patients with HSK, is a frequent indication for surgery. Due to the caudal and medial locations of calyces and the abnormal anterior position of the kidney, extracorporeal shock wave lithotripsy has shown a relatively low success rate in treating HSK. Percutaneous nephrolithotomy has also been associated with major complications in anomalous kidneys. Advances in laparoscopic instrumentation and techniques have made laparoscopic surgery a promising alternative for stone treatment in HSK. This report describes a 61-year-old woman who presented initially with recurrent UTI unresponsive to multiple courses of antibiotics. Urine cultures were positive for Escherichia coli. A computed tomography scan showed a right HSK with multiple renal stones (35 mm in the right lower pole with eight stones 2-4 mm in size), along with severe hydronephrosis. The patient was treated successfully with laparoscopic nephrolithotomy, indicating that laparoscopy is an effective and safe approach in the treatment of renal stones >2 cm in HSK.

## Introduction

Horseshoe kidney (HSK) is the most common congenital type of renal fusion anomaly, with an incidence of 1:400 individuals, and is more prevalent in men than in women [1]. HSK, which is frequently asymptomatic, is characterized by malrotation of the kidney, altered blood supply, and, in 30% of patients, a tendency to obstruct the pelvi-ureteric junction [2]. Fusion occurs in the lower part of the kidney in 95% of patients and tends to be detected incidentally [3]. However, HSK may present with complications, such as urinary tract infections (UTI), stone formation, and obstruction [2,3].

HSK formation is due to the failure of normal anatomical rotation of the kidneys during embryological development. Malrotation results in the pelvis being situated in front of the kidneys and the ureter entering the pelvis at a higher level. These anomalies increase the risk of formation of kidney stones that cannot be passed spontaneously.

Anatomical abnormalities in patients with HSK present a challenge in the treatment of kidney stones. For instance, due to the caudal and medial locations of calyces and the abnormal anterior position of the kidney, extracorporeal shock wave lithotripsy (ESWL) is less successful in HSKs than in normal kidneys [4] Percutaneous nephrolithotomy (PCNL) is also associated with major complications in the anomalous kidney [5]. Improvements in laparoscopic instrumentation and techniques and in surgical experience have made laparoscopic surgery a promising alternative for stone treatment in HSK [6].

This report describes a 61-year-old woman who presented initially with recurrent UTI unresponsive to multiple courses of antibiotics. Computed tomography (CT) scanning revealed a right-side HSK with multiple renal stones. The patient was treated successfully with laparoscopic nephrolithotomy.

## Case presentation

A 61-year-old fit and healthy woman presented initially to her local general practitioner with recurrent UTIs. Treatment with courses of multiple antibiotics was unsuccessful. An ultrasound scan of the abdomen showed a duplex right kidney with multiple stones associated with moderate-severe dilatation of the right pelvicalyceal system. She was referred to our department for further evaluation.

**Figure 1:**
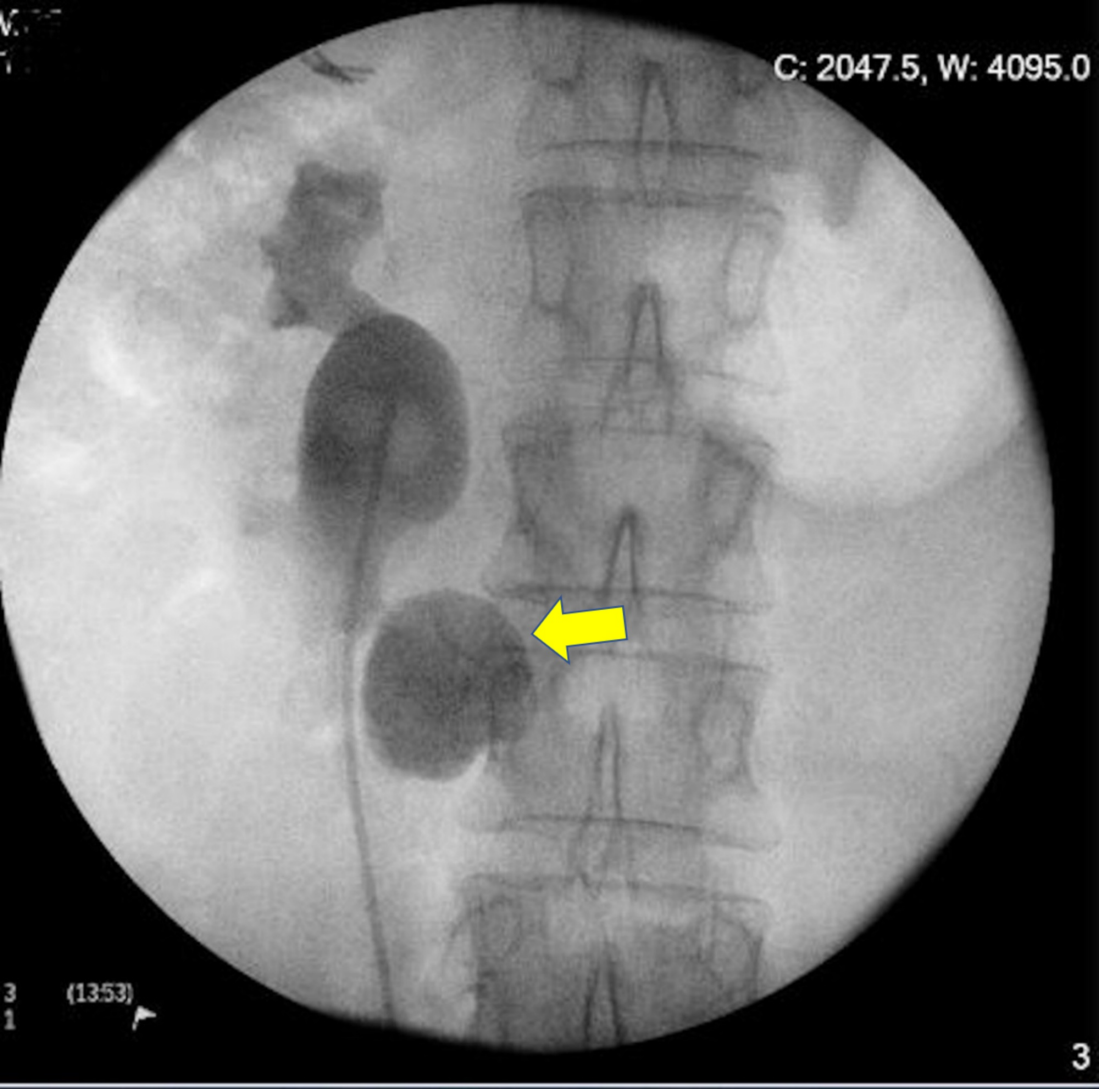
Intravenous pyelogram shows a large stone in the lower pelvis of the right HSK HSK, horseshoe kidney

Her previous medical history included a cholecystectomy, but no significant medical issues. Her physical examination was unremarkable, and her body mass index was 26 kg/m^2^. Renal function tests showed a normal creatinine concentration of 61 µmol/L and an estimated glomerular filtration rate of 87 mL/min/1.73 m^2^; urinalysis showed leucocytosis and erythrocytosis; and a urine culture confirmed the presence of Escherichia coli bacteria, for which she was treated with appropriate antibiotics. A urinary radiogram (kidney, ureter, and bladder [KUB]) showed a 3-cm opacity in the lower pole of her right kidney, and an intravenous pyelogram showed a functioning HSK (Figure 1).

**Figure 2:**
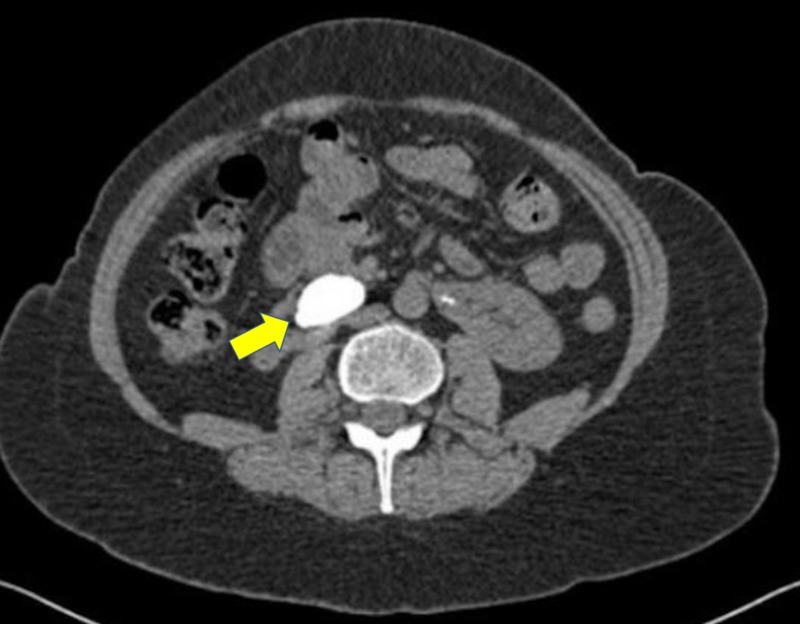
Non-contrast CT scan demonstrating a large calculus in the right HSK HSK, horseshoe kidney

**Figure 3:**
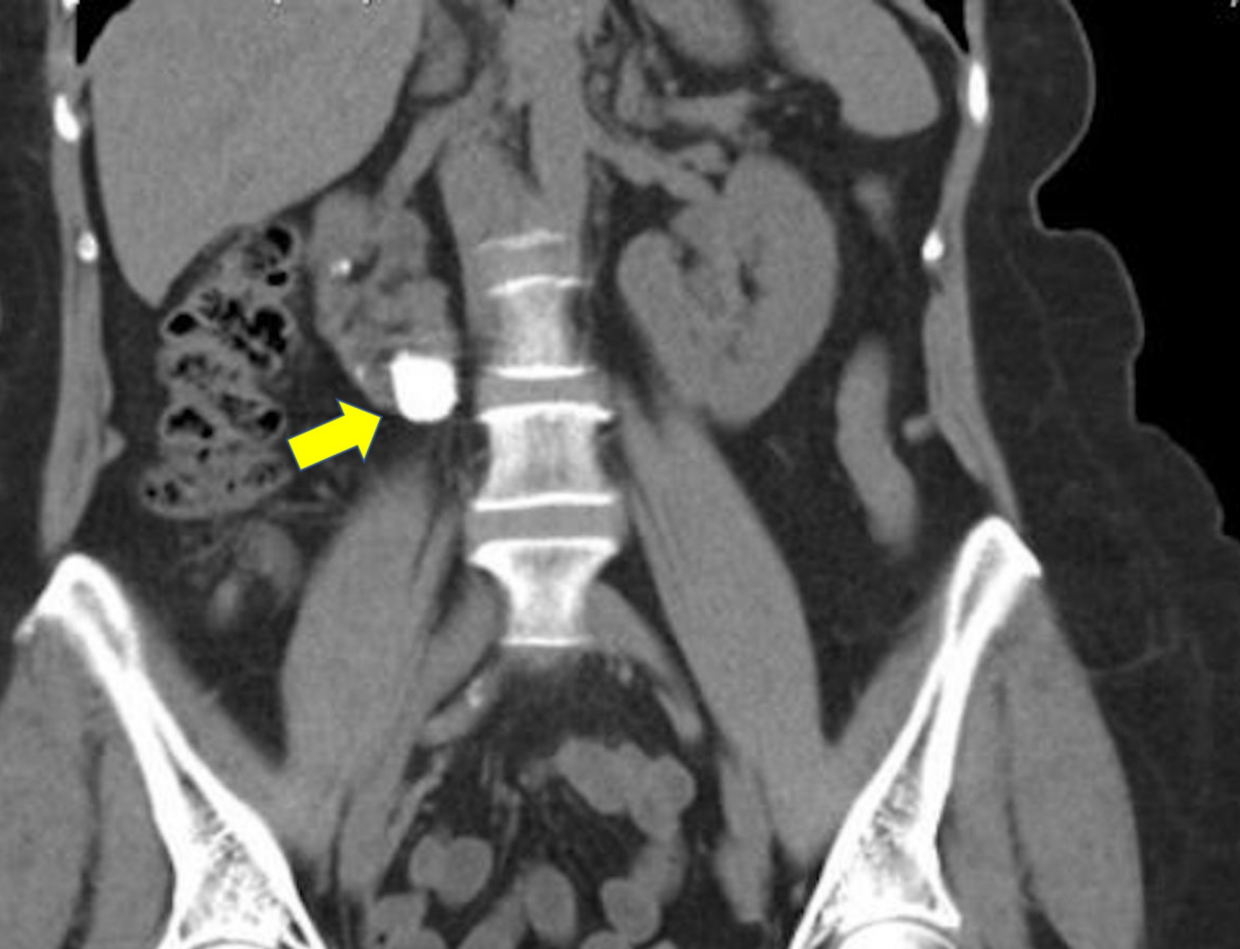
A coronal view of non-contrast CT scan showing the lower pole calculus

A CT KUB showed a right HSK with multiple renal stones, measuring 35 mm in the right lower pole, and consisting of eight stones, 2-4 mm in size. The lower poles of both kidneys were fused in front of the aorta, and the left renal collecting system was normal. Also, the right kidney appeared severely hydronephrotic, with a large opacity extending from the renal pelvis to the lower pole and no flow of contrast through the right ureter (Figures 2, 3).

A 99mTc-dimercaptosuccinic acid scintigraphy scan showed split function of the kidneys, 73.8% on the left side and 26.2% on the right side. A double-J (DJ) stent was therefore implanted on the right side to relieve the obstruction.

The multidisciplinary team meeting recommended a laparoscopically assisted PCNL; however, after a deep discussion with the patient and a wider consideration of various risks and benefits, we opted to proceed with an elective laparoscopic nephrolithotomy.

Surgical technique

**Figure 4:**
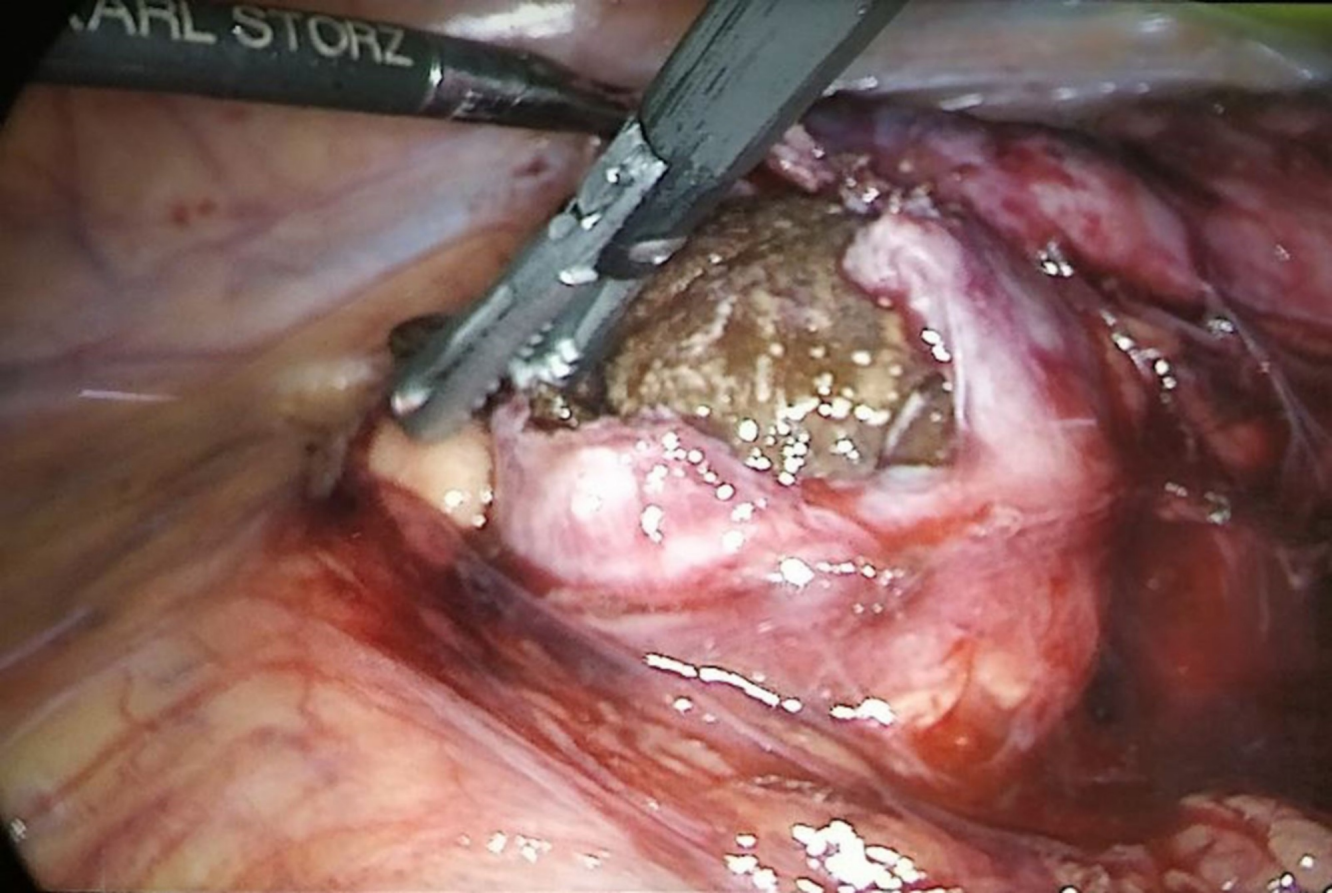
Intraoperative laparoscopic image showing the calculus being removed from the thinned lower kidney pole

The patient was placed in the left lateral position under general anesthesia and administered prophylactic gentamycin on induction. A 10-mm Hassan’s port and two 12-mm ports were inserted, and carbon dioxide was insufflated into her peritoneal cavity to create pneumoperitoneum. The right kidney and isthmus of the HSK were identified, and the colon was medially mobilized. The stone-containing calyx was identified under X-ray guidance. The thinned-out cortex was opened (Figure 4), and the stone was removed. Large pieces of the stone were placed in a bag and retrieved, and small fragments were suctioned out. Cavity edges were sutured with 4-0 Vicryl. Collagen foam was applied for hemostasis. A drain was inserted, and all ports were closed.

The total duration of hospitalization with drain placement was three days. No procedural complications were observed, and a complete stone-free status was achieved. Postoperative radiographs showed no evidence of residual stone fragments. The DJ ureteric stent was removed four weeks after the procedure. 

## Discussion

HSK results from a defect in embryogenesis occurring between the fourth and eighth weeks of gestation. The lower poles of both kidneys fuse during this anomalous developmental phase alongside the inferior mesenteric arteries, which arrests their ascent to their normal position. Kidney fusion may be due to a fibrous tissue band or thick functional renal tissue. Because the kidneys, ureters, and pelvis are in abnormal positions, patients with HSK may clinically present with infection, obstruction, calculi, or even a tumor [7].

Urolithiasis is the most common complication of HSK, with 20% to 60% of these stones requiring surgical treatment. The congenital anomalies of HSK make the treatment of stones more challenging, necessitating different anatomical approaches. The treatment options for stones in HSK include PCNL, ESWL, ureterorenoscopy (URS), laparoscopy, and open surgery. Although PCNL and ESWL are preferred, their results have been inconsistent and suboptimal in some patients. For example, the post-ESWL stone-free rate in patients with HSK ranges from 25% to 75%. ESWL has been reported safe in patients with stones ≤2 cm whose urinary flow is not obstructed [8,9]. Because our patient had a stone larger than 2 cm, ESWL was not preferred in our case.

Laparoscopy has been used alone or in conjunction with other endoscopic procedures for the treatment of stones in anomalous kidneys. The use of laparoscopy to manage stones in patients with HSK was first reported in 2004 [10].

Although PCNL is the treatment of choice for stones >2 cm, this method is associated with serious complications in patients with HSK. These complications are associated with the abnormal anatomy in patients with HSKs, including the altered position of the kidneys, the aberrant characteristics of the blood supply, and the anomalous communications between ureters [11].

The patient described in this study had a relatively large calculus, stone size 3.5 cm, making URS less feasible. Laparoscopic nephrolithotomy was preferred not only because of the large stone size but also because of the absence of an extrarenal pelvis and the presence of aberrant vessels crossing the renal pelvis. Laparoscopic nephrolithotomy was therefore considered a better option, as it can provide easy access to stones and offer complete removal of calculi.

The first study describing the outcomes of laparoscopic nephrolithotomy in patients with staghorn calculi was reported in 1994 [12]. A more recent meta-analysis found that PCNL and laparoscopic nephrolithotomy were safe, effective, and reliable treatment options in the removal of large single stones >2 cm in size, with PCNL and laparoscopic nephrolithotomy having stone-free rates of 98% and 89%, respectively [13]. This meta-analysis suggested that laparoscopic nephrolithotomy is safe and effective for treating >2 cm renal stones, particularly in patients with ureteropelvic junction stenosis or anomalous kidneys. The results in our patient support these findings and demonstrate the safety and effectiveness of this treatment option. However, more cases are to be considered to support the results further. 

## Conclusions

HSK is the most common type of renal anomaly, with urolithiasis being its most frequent complication. Various treatment options and surgical procedures are available for the management of stones in HSK, but these treatment modalities have been associated with serious complications and suboptimal results. Laparoscopic nephrolithotomy was successful in our patient, suggesting that laparoscopy is an effective and safe approach to treat renal stones >2 cm in patients with HSK. However, large-scale adoption of laparoscopic nephrolithotomy in the management of stones in HSK can assist in determining the safety and the effectiveness of this procedure.
